# Response of Energy Reserves in Entomopathogenic Nematodes to Drought-Stress and Expression Analysis of Energy Metabolism-Related Genes in Arid Areas

**DOI:** 10.3390/insects17010022

**Published:** 2025-12-23

**Authors:** Xia Wu, Wenliang Li, Tingwei Zhang, Hong Chen, Wende Zhang, Xingduo Wang, Xiujuan Qian

**Affiliations:** Biocontrol Engineering Laboratory of Crop Diseases and Pests of Gansu Province, College of Plant Protection, Gansu Agricultural University, Lanzhou 730070, China

**Keywords:** entomopathogenic nematodes, low-humidity stress, energy reserves, metabolism-related gene

## Abstract

The free-living infective juveniles of entomopathogenic nematodes (EPNs) are critical biological control agents against insect pests. The field efficacy of EPNs is largely determined by their tolerance to low-humidity stress, a trait closely linked to the mobilization of their energy reserves. This study aims to investigate how varying levels of humidity stress influence energy reserve dynamics in two EPNs, *Steinernema kraussei* 0657L and *Heterorhabditis brevicaudis* 0641TY, and their relationship with the survival rate and pathogenicity. The results demonstrated that lipids were the predominant energy reserve, followed by proteins and sugars. Notably, neutral lipid constituted approximately 3% of the total lipid content. Among sugars, soluble sugar levels were the highest, followed by glycogen and trehalose. Exposure to low-humidity stress resulted in increased levels of protein, total lipid, glycogen, and trehalose in nematodes. These increases were more pronounced in *S. kraussei* 0657L, a highly drought-resistant strain, compared to *H. brevicaudis* 0641TY. Furthermore, the accumulation of protein, total lipid, and trehalose was negatively correlated with survival rate and pathogenicity. However, positive correlations were observed between trehalose and both total lipids and soluble sugars. Furthermore, transcriptome analysis revealed that under low-humidity stress, *S. kraussei* 0657L exhibited an enrichment of differentially expressed genes (DEGs) involved in glycolysis/gluconeogenesis, fatty acid metabolism, and glycerophospholipid metabolism pathways. This indicates that *S. kraussei* 0657L regulated energy metabolism to adapt to low-humidity stress. These findings provide insights into the mechanisms underlying drought resistance in EPNs and offer an experimental basis for their application in arid environments.

## Simple Summary

Entomopathogenic nematodes (EPNs) (genera: *Steinernema*, *Heterorhabditis*, and *Oscheius*), which kill insect hosts with the aid of symbiotic bacteria, are used widely as biocontrol agents in agricultural pest control because of their advantages of active search for pests, wide host range, and safety to humans, livestock, and the environment. In particular, they have good control effects on boring pests and soil pests. As a novel type of biological agent, the energy reserve capacity and low-humidity adaptability of EPNs in the natural environment are directly linked to their pest control efficiency. In this study, we measured energy reserves, biological traits, and behavioral characteristics of two EPN species under low humidity conditions, and performed transcriptome analysis on drought-resistant strains. This approach offers a new perspective for elucidating the mechanisms of drought resistance in EPNs.

## 1. Introduction

Agricultural pests cause economic damage to crop and grain production and, therefore, are key control targets. The use of pesticides is the primary method of pest control, but concerns about their adverse effects on human health and the environment have driven research into more sustainable alternatives. Consequently, environmentally friendly pest control methods have gained increasing attention. Biological control is an effective method to reduce pesticide residues, and entomopathogenic nematodes (EPNs), such as *Steinernema* and *Heterorhabditis*, have proven to be effective biocontrol agents of pests, as they have shown control effects on more than 250 species of concealed pests [1,2,3,4]. To date, more than 100 species of EPNs have been studied; only about 10 to 13 species have been commercialized as alternative pesticides for biological control [5,6,7].

Nematodes in the infective juvenile (dauers) stage actively search for insect hosts in the soil and enter through wounds or natural orifices, such as the mouth, spiracle, and anus [8]. After entry, the symbiotic bacteria (e.g., *Xenorhabdus* or *Photorhabdus* spp.) in their intestine are released into the insect’s hemocoel, which propagates and produces toxic secondary metabolites in 24–48 h [9]. However, infective juveniles (IJs, developmentally arrested third-stage larvae) are highly sensitive to environmental factors such as humidity, UV-B, and temperature [10,11,12]. Among these, humidity is considered the most critical factor influencing the control efficacy of EPNs. Soil moisture in their habitat is affected by irrigation and local climate, which can create adverse conditions for nematodes, such as desiccation, high osmotic pressure, and hypoxia [13]. Additionally, humidity is a major limiting factor for the application of EPNs against foliar insect pests [14]. Notably, the free-living IJs of EPNs are non-feeding and rely exclusively on stored energy reserves for survival [15]. Thus, learning how IJs resist anhydrobiosis by mobilizing energy substances can provide valuable insights into their capacity to endure drought conditions.

The energy reserves of infective juveniles (IJs) form the foundation of their physiological and behavioral adaptations to stress, influencing both individual survival and field control efficacy [16]. For example, the *Steinernema carpocapsae* CB-16 strain exhibited a high survival rate and the ability to resist anhydrobiosis after 9 h of exposure to low humidity [17]. Similarly, cold acclimation in *S. litorale* induced changes in soluble sugar, lipid, and glycogen contents, enhancing its cold tolerance [18]. Additionally, *Heterorhabditis* sp. (HP2) showed the highest total lipid content, penetration rate, and virulence among six tested strains [19]. Moreover, drought exposure reduced oxygen consumption, water content, and total sugar and glycogen levels, while crude fat and fatty acid content remained unchanged [20]. Glycerol and trehalose have also been identified as critical for nematode adaptation to marginal habitat [21]. These findings suggest a clear connection between energy reserves and the biological characteristics of nematodes. Furthermore, reductions in neutral lipid and glycogen reserves in *S. carpocapsae* and *S. feltiae* were shown to weaken their pathogenicity [22]. Stress-induced changes in energy reserves directly affect the behavior of entomopathogenic nematodes (EPNs), which adopt one of two feeding strategies: cruising or ambushing. Cruisers primarily consume sugars, experiencing a rapid decline in energy reserves and an increased metabolic rate, while ambushers exhibit a slower decline in lipids [13,23,24]. These factors interact to regulate stress tolerance in nematodes. Therefore, it is essential to investigate the changes in energy reserves of IJs under stress conditions.

The mutual transformation of energy reserves supports the homeostasis of EPNs under adverse stress conditions, thereby enhancing their adaptability to adverse stress [25]. The maintenance of this balance is governed by complex molecular mechanisms. Transcriptomic analysis enables the analysis of gene regulation and molecular mechanisms at the transcriptional level, providing a valuable tool for investigating the stress resistance mechanisms of EPNs from a genetic perspective [26]. For instance, Yarri et al. utilized transcriptomic analysis to investigate the molecular and physiological mechanisms underlying dehydration and heat stress tolerance in various nematode strains [16]. A comparative transcriptomic analysis conducted during the early stage of oxidative stress induction (4 h) in two *Heterorhabditis bacteriophora* inbred lines revealed the mechanisms underlying the response of dauer longevity to oxidative stress tolerance [27]. Despite these numerous physiological and molecular studies, the links between metabolite dynamics and gene expression, specifically under low-humidity stress in EPNs, remain poorly understood.

The climate of Gansu Province in China is primarily arid, with precipitation concentrated in summer, accounting for about 70% of the annual total. Previous studies indicated that *Steinernema kraussei* 0657L was more resistant to low-humidity stress than *H. brevicaudis* 0641TY [28]. In the present study, the two strains were selected due to their strong resistance to stressful conditions, as found in Gansu Province. We compared the responses of energy substances in *S. kraussei* 0657L and *H. brevicaudis* 0641TY to low-humidity stress and analyzed the correlation between these substances and the survival rate, as well as the pathogenicity of different nematode strains under such conditions. Additionally, the transcriptome of *S. kraussei* 0657L, recognized for its strong drought resistance, was analyzed under varying levels of relative humidity. This analysis further explored the molecular mechanisms of energy metabolism that enable *S. kraussei* 0657L to adapt to low-humidity stress.

## 2. Materials and Methods

### 2.1. Nematodes

*Steinernema kraussei* 0657L and *Heterorhabditis brevicaudis* 0641TY were provided by the Insect Ecology Laboratory of Gansu Agricultural University and were reared on the last-instar larvae of the wax moth, *Galleria mellonella* (Linnaeus) (White-trap), in an LED cold-light artificial intelligence climate box (Shanghai Bowen Industrial Co., Ltd., Shanghai, China) under a 14:10 h light/dark cycle, at 25 ± 1 °C, 85% ± 10% relative humidity. The nematode suspension was stored in a refrigerator (Qingdao Haier Co., Ltd., Qingdao, China) for 3–4 weeks at 4 °C.

### 2.2. Determination of Survival Rate and Pathogenicity of S. kraussei 0657L and H. brevicaudis 0641TY After Exposure to Low Humidity

A total of 3000 IJs of each strain were used. The samples were briefly centrifuged (800× *g*, 25 °C), and the supernatant was discarded. The concentration of the samples was adjusted to 30 IJs/μL, and 100 μL of the nematode suspension was transferred to a 9 cm Petri dish lined with dry filter paper. The nematodes were carefully dispersed to prevent the formation of tight coils. Subsequently, the nematodes were placed into a small desiccator (25 °C) containing a saturated salt solution to maintain relative humidity (RH) levels of 60% and 30%, following the method described by Gaugler [28]. After low-humidity treatment, twelve IJs were randomly selected from each group for observation, with untreated nematodes serving as the control. The nematodes were cultured at 25 °C, and their survival was monitored every 20 min. Nematodes were classified as dead if they exhibited a straight body posture and showed no response to mechanical stimulation. Survival rates were calculated until all the IJs were dead. Each treatment was repeated at least five times, and then Kaplan–Meier lifespan analysis was performed.

The pathogenicity of the two EPN species was determined using the improved ONE ON ONE [28]. To determine this, the late instar larvae of *G. mellonella* were used. They were placed into a 24-well plate (one larva per well) with two layers of filter paper. Then 30 IJs of *S. kraussei* 0657L and *H. brevicaudis* 0641TY, which had been exposed to low-humidity stress, were added individually to each well. A volume of 50 μL of water was added, and the plate was placed inside a climate box (25 ± 1 °C). The control setup was IJs stored in distilled water. There were 30 *G. mellonella* per treatment, and the mortality was recorded at 8, 16, 24, 32, 40, 48, and 56 h after treatment. All experiments were performed with three independent biological replicates for each treatment.

### 2.3. Determination of Energy Substances

The contents of protein, glycogen, and trehalose were measured using the Protein Content Assay Kit (Solarbio^®^ BC3185), Glycogen Content Assay Kit (Solarbio^®^ BC0340), and Trehalose Content Assay Kit (Solarbio^®^ BC0330), respectively, following the manufacturer’s instructions (Solarbio, Beijing, China).

To determine the content of soluble sugar, total lipid, and neutral lipid, IJs exposed to low-humidity stress for a median lethal time (LT_50_) were collected. Approximately 1000 IJs per replicate were transferred into 1.5 mL nuclease-free tubes. Each treatment was performed in triplicate. Subsequently, 50 μL of buffer, containing 100 mM KH_2_PO_4_ (potassium dihydrogen phosphate), 1 mM DTT (dichlorodiphenyltrichloroethane), and 1 mM EDTA (ethylenediaminetetraacetic acid), pH = 7.4, was added to each tube, and the samples were ground using a high-speed mini-Beadbeater homogenizer (BioSpec Products, Inc., Bartlesville, OK, USA) at 11,000 rpm.

#### 2.3.1. Soluble Sugar

The soluble sugar content was quantified according to the methodologies described by Liu [29].

To measure the soluble sugar, the supernatant of the homogenate was transferred to 2 mL nuclease-free tubes, and 20 μL of 20% sodium sulfate solution was added to promote protein precipitation and facilitate sugar extraction. The analyzed samples were then centrifuged (10,000 r/min, 4 °C) for 15 min in a 1500 μL chloroform-methanol mixture (Vc:Vm = 1:2). Volumes of 450 μL of the supernatants were transferred into 2 mL nuclease-free tubes and completely evaporated at room temperature. Then 30 μL of distilled water was added to each tube, followed by 720 μL of anthrone reagent (80% H_2_SO_4_, 1.42 g/L). After 15 min at room temperature, the reaction was heated in a boiling water bath for 15 min before being cooled on ice. The reaction solution was transferred to a 96-well enzyme panel, and the absorbance was measured at 630 nm. Glucose was used as the standard curve. The aforementioned analytical procedure was performed on six separate occasions consistently.

#### 2.3.2. Total Lipid and Neutral Lipid

The methodology for determining the content of total lipid and neutral lipid followed procedures delineated by Lv et al. [30].

Total lipid: The supernatant of the test sample (100 μL) was transferred into nuclease-free tubes, and the solvent was evaporated at 90 °C. After, 10 μL of 98% H_2_SO_4_ was added and heated in a water bath to 90 °C for 2 min, then cooled on ice. Then vanillin (68% H_2_PO_3_, 1.2 g/L) was added to the sample. Finally, after 15 min, the OD value was determined at 525 nm. Triolein was used as the standard, and different concentrations of that were used to construct a standard curve. The aforementioned analytical procedure was performed on six separate occasions consistently.

Neutral lipid: The supernatant (500 μL) was transferred into 1.5 mL nuclease-free tubes and heated to 90 °C until all the solvent had completely evaporated. Then 1 mL of chloroform was added to dissolve the lipid. After, 200 mg of dry silicon acid (200 °C, 24 h) was then added to each sample to solidify polar lipids. Finally, 100 μL of the supernatant was centrifuged (10,000 r/min, 4 °C) for 10 min. The OD value was determined at 525 nm. Triglyceride was used as the standard, with varying concentrations used to construct the standard curve.

### 2.4. Correlation Analysis of Energy Substances in Entomopathogenic Nematodes with Survival Rate and Pathogenicity

Pearson correlation analysis was performed to evaluate the relationship between energy reserves (protein, soluble sugar, glycogen, total lipid, neutral lipid, and trehalose), survival rate, and pathogenicity. The analysis included 9 independent observations (*n* = 9), representing 3 replicates from each of the 3 treatment groups. Prior to the analysis, we verified the assumption of normality using the Shapiro–Wilk test (*p* > 0.05) and checked for outliers using scatter plots. The results demonstrated a robust linear relationship with statistically significant *p*-values (*p* < 0.05), indicating that the sample size was sufficient to detect the strong correlation between energy reserves and survival rates in this experimental context. All statistical analyses were conducted using IBM SPSS Statistics for Windows, Version 23.0 (IBM Corp., Armonk, NY, USA).

### 2.5. RNA-Seq Analysis and RT-qPCR Verification

In this study, *S. kraussei* 0657L, with strong adaptability to low-humidity stress, was selected for subsequent molecular study on the mechanism of drought resistance. Total RNA was extracted from nematodes in the control group and the low-humidity treatment group using Trizol Reagent [31]. For each sample, approximately 1000 IJs were collected. Three independent biological replicates were performed for each treatment group. PrimeScriptTM RT reagent Kit and TB Green^®^ Premix Ex TaqTM II (Shanghai BioScience Co., Ltd., Shanghai, China) were used for gDNA eraser and quantitative RT-PCR. The primers were designed using the NCBI online website (https://www.ncbi.nlm.nih.gov/tools/primer-blast/, 28 September 2025). According to the list in Table 1.

The RNA-seq in this study was commissioned by Beijing Biomarker Technologies Co., Ltd. (Beijing, China) to complete the library construction, and the cDNA library was sequenced using the Illumina NovaSeq high-throughput sequencing platform (Beijing, China) [32].

### 2.6. Differential Expression Analysis

DESeq2 [33] was employed to identify differentially expressed genes (DEGs) across the sample groups, yielding DEG sets specific to the two conditions. Differential expression was determined using a false discovery rate (FDR) threshold of <0.05 and a fold change (FC) of ≥1.5 as screening criteria.

### 2.7. GO and KEGG Pathway Enrichment Analysis

The differentially expressed genes (DEGs) were analyzed and enriched using the Gene Ontology (GO) and Kyoto Encyclopedia of Genes and Genomes (KEGG) databases. Functional annotations were assigned to the DEGs, and clustering analyses of GO components and KEGG pathways were performed to identify relevant DEGs.

### 2.8. Construction of Protein–Protein Interaction (PPI) Network and Expression Analysis of DEGs in S. kraussei 0657L Under Different Humidity Stress

The STRING database (https://cn.string-db.org/, 31 October 2025) was utilized to construct the interaction network of differentially expressed genes (DEGs) by integrating the results of DEGs with interaction pairs provided by the database [34]. Cytoscape version 3.6.1 was employed for the visualization and editing of DEG protein interactions.

### 2.9. Statistical Analyses

Data compilation and statistical analyses were performed using Microsoft Excel 2016, Origin 2022, and SPSS 23.0 (IBM Corp., Armonk, NY, USA). The relative expression of each gene for RT-qPCR analysis was calculated using the 2^−∆∆Ct^ method. For longevity and stress resistance assays, survival curves were compared using the Log-rank (Mantel–Cox) test. For desiccation tolerance assays, the median lethal time (LT_50_) and 95% confidence intervals (CIs) were calculated using Probit analysis; significance was determined based on non-overlapping 95% CIs. Prior to parametric comparisons, the normality of the data was verified using the Shapiro–Wilk test, and homogeneity of variances was assessed using Levene’s test. Data satisfying these assumptions were analyzed using Student’s *t*-test for comparisons between two groups or one-way analysis of variance (ANOVA) followed by Tukey’s honestly significant difference (HSD) test for multiple comparisons among treatments. Pearson correlation analysis was conducted to evaluate the relationship between variables after confirming normal distribution. All data are expressed as mean ± standard error (SE) from at least three replicates. Differences were considered statistically significant at *p* < 0.05.

The rate of change in energy substances was calculated using the following formula: R = ∣C_treatment_ − C_control_∣/T. Where represents the rate of change (mg·g^−1^·h^−1^), C_treatment_ and C_control_ represent the content of the substance in the treatment and control groups, respectively, and T is the exposure time in hours (h).

## 3. Results

### 3.1. Survival Rate and LT_50_ of Two Species of Entomopathogenic Nematodes After Low Humidity

**Figure 1:**
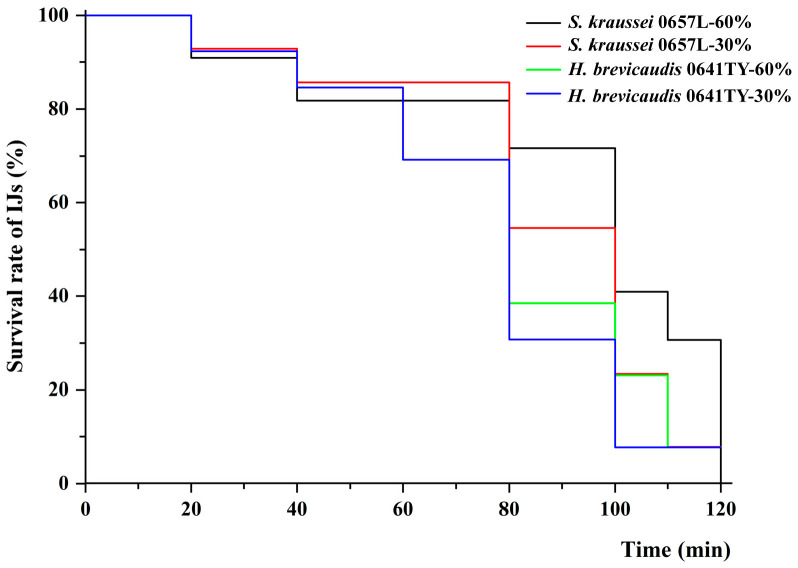
Effects of different relative humidity on the lifespan of two entomopathogenic nematode species. IJs were treated at relative humidity of 60% and 30% at 25 °C. The number of dead nematodes was recorded every 20 min until all the nematodes had died, and a survival curve was plotted.

The two entomopathogenic nematode (EPN) species exhibited significant differences in their ability to withstand low-humidity conditions (Figure 1). *S. kraussei* 0657L demonstrated greater tolerance to low humidity compared to *H. brevicaudis* 0641TY. After 100 min of exposure to 60% humidity, the survival rate of *H. brevicaudis* 0641TY was 27.19%, which dropped to 0% when exposed to 30% humidity. In contrast, the survival rate of *S. kraussei* 0657L under 60% humidity was significantly higher than that of *H. brevicaudis* 0641TY. When the exposure time was prolonged to 120 min, the survival rate of *S. kraussei* 0657L under 30% RH and *H. brevicaudis* 0641TY under 60% RH were both reduced to 0%. However, *S. kraussei* 0657L maintained a significantly higher survival rate (66.24%) under RH 60% compared to *H. brevicaudis*.

The survival rates of the two strains were inversely proportional to the duration of the exposure to the humidity treatments, decreasing as exposure time increased. The median lethal time (LT_50_) for each nematode species under 60% and 30% humidity conditions was 112 and 75 min for *S. kraussei* 0657L and 75 and 67 min for *H. brevicaudis* 0641TY (Table 2).

### 3.2. The Pathogenicity of EPNs After Exposure to Different Humidities

The pathogenicity of *S. kraussei* 0657L and *H. brevicaudis* 0641TY decreased with a reduction in humidity; lower relative humidity resulted in significantly reduced host mortality rates (Table 3). For *S. kraussei* 0657L, humidity had a significant effect on host mortality (F_2,6_ = 15.823, *p* < 0.05). Under control conditions (100% RH), host mortality reached 100% at 48 h. At 60% RH, mortality remained high (94.45% at 56 h) and was not significantly different from the control (*p* > 0.05). However, exposure to 30% RH significantly reduced pathogenicity to 66.67% (*p* < 0.05). For *H. brevicaudis* 0641TY, the pathogenicity was also markedly suppressed by low humidity (F_2,6_ = 24.105, *p* = 0.001). At 30% RH, the mortality rate at 56 h dropped to 58.33%, representing a significant decrease compared to the control (*p* < 0.05). Comparison between the two strains at the same humidity levels (*t*-test) showed that *S. kraussei* 0657L generally exhibited higher pathogenicity than H. brevicaudis 0641TY, particularly under stress conditions.

### 3.3. Effect of Different Humidity Stress on Energy Substances in H. brevicaudis 0641TY and S. kraussei 0657L

**Figure 2:**
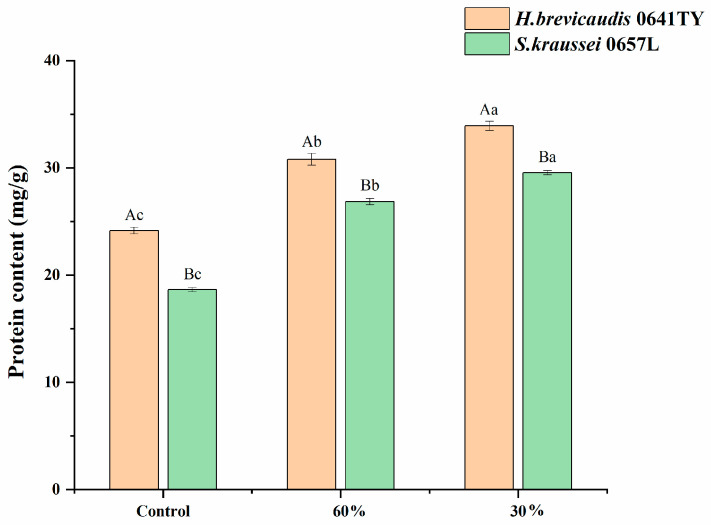
Protein content (mg/g) in *H. brevicaudis* and *S. kraussei* after exposure to different humidity levels (control means IJs were stored in distilled water, RH 100%; the measured protein content was the content of all IJs after low-humidity stress). Different lowercase letters indicate significant differences between levels of relative humidity for the same nematode strain at a 0.05 significance level. Different capital letters indicate significant differences between nematode strains under the same relative humidity at a 0.05 significance level (Tukey’s test).

Protein. Low-humidity stress led to an increase in protein content in both *S. kraussei* 0657L and *H. brevicaudis* 0641TY (Figure 2). The protein content in *H. brevicaudis* 0641TY was consistently and significantly higher than that of *S. kraussei* 0657L across all humidity levels tested (*p* < 0.05). Under 60% RH, the protein content of *H. brevicaudis* 0641TY reached 30.8 mg/g, which was significantly higher than the control *(p* < 0.05). At 30% RH, the protein content increased further to 33.9 mg/g, representing a significant increase of 9.76 mg/g compared to the control (*p* < 0.05).

**Figure 3:**
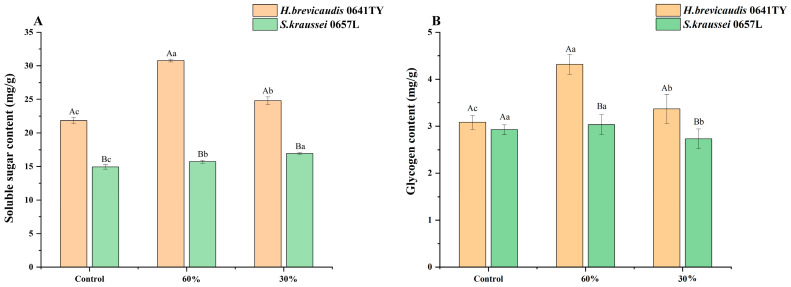
(**A**) Soluble sugar and (**B**) glycogen in *H. brevicaudis* and *S. kraussei* after exposure to different humidity levels (control means IJs were stored in distilled water, RH 100%; the measured content was the content of all IJs after low-humidity stress). Different lowercase letters indicate significant differences between levels of relative humidity for the same nematode strain at a 0.05 significance level. Different capital letters indicate significant differences between nematode strains under the same relative humidity at a 0.05 significance level (Tukey’s test).

Soluble sugar and glycogen. Soluble sugar and glycogen are widely associated with stress resistance. Exposure to varying levels of humidity stress led to the accumulation of soluble sugar and glycogen in IJs (Figure 3). The soluble sugar content in *H. brevicaudis* 0641TY was significantly higher than that of *S. kraussei* 0657L (*p* < 0.05), both before and after treatment (Figure 3A). Under low-humidity stress, soluble sugar in *H. brevicaudis* 0641TY accumulated rapidly. The rate of increase was 7.15 mg·g^−1^·h^−1^ at 60% RH and 2.65 mg·g^−1^·h^−1^ at 30% RH. In contrast, the soluble sugar content in *S. kraussei* 0657L significantly increased under low-humidity stress (*p* < 0.05), rising from 14.9 mg/g in the control group to 15.7 mg/g at 60% RH and 16.9 mg/g at 30% RH.

Differing humidity levels induced differential changes in glycogen content between the two nematodes (Figure 3B). Prior to the humidity treatments, there was no significant difference in the glycogen content between *H. brevicaudis* 0641TY and *S. kraussei* 0657L (*p* > 0.05). Under 60% RH, *H. brevicaudis* 0641TY exhibited a significant accumulation of glycogen, peaking at 4.32 mg/g, which was significantly higher than the untreated control (*p* < 0.05). In contrast, for *S. kraussei* 0657L (Figure 3B), glycogen content at 60% RH did not differ significantly from the control (*p* > 0.05). At 30% RH, the glycogen level significantly decreased compared to both the control and the 60% RH group (*p* < 0.05). This ability to maintain stable glycogen reserves without depletion suggests that *S. kraussei* 0657L possesses a more robust tolerance mechanism.

**Figure 4:**
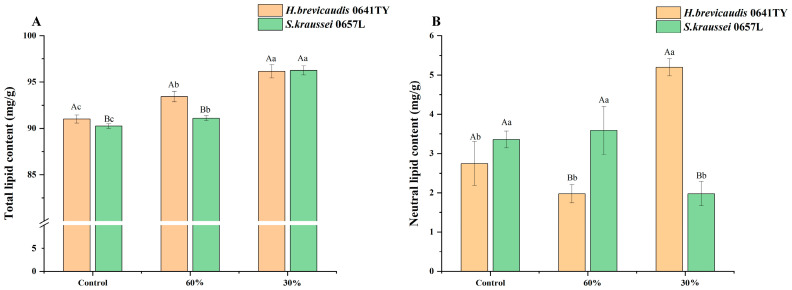
(**A**) Total lipids and (**B**) neutral lipids in *H. brevicaudis* and *S. kraussei* after exposure to different humidity levels (control means IJs were stored in distilled water, RH 100%; the measured content was the content of all IJs after low-humidity stress). Different lowercase letters indicate significant differences between levels of relative humidity for the same nematode strain at a 0.05 significance level. Different capital letters indicate significant differences between nematode strains under the same relative humidity at a 0.05 significance level (Tukey’s test).

Total lipid and neutral lipid. Total lipids and neutral lipids in nematodes are closely associated with their high pathogenicity. The results revealed that varying levels of humidity stress increased lipid content in both nematodes (Figure 4). The total lipid content in the two nematodes increased gradually as RH decreased (Figure 4A). The maximum lipid content in *H. brevicaudis* 0641TY was 96.15 mg/g at 30% RH, which was significantly different from the control (*p* < 0.05) and the level observed at 60% RH. Similarly, the maximum lipid content in *S. kraussei* 0657L was 96.25 mg/g at 30% RH, which was significantly higher than the content in the control and at 60% RH (*p* < 0.05).

Our results also indicated that varying humidity stress increases the neutral lipid content in the two nematodes (Figure 4B). There was no significant difference in neutral lipid content between *H. brevicaudis* 0641TY and *S. kraussei* 0657L in the control (*p* > 0.05). However, the neutral lipid content in *H. brevicaudis* 0641TY at 60% RH was 1.97 mg/g, which was significantly lower than that of the control (2.74 mg/g, *p* < 0.05). At 30% RH, the content increased to 5.20 mg/g. In contrast, the neutral lipid in *S. kraussei* 0657L was 3.59 mg/g at 60% RH, which was not significantly different from that of the control (*p* > 0.05); however, at 30% RH, the lipid content decreased to 1.82 mg/g, which was significantly lower than the control (*p* < 0.05).

**Figure 5:**
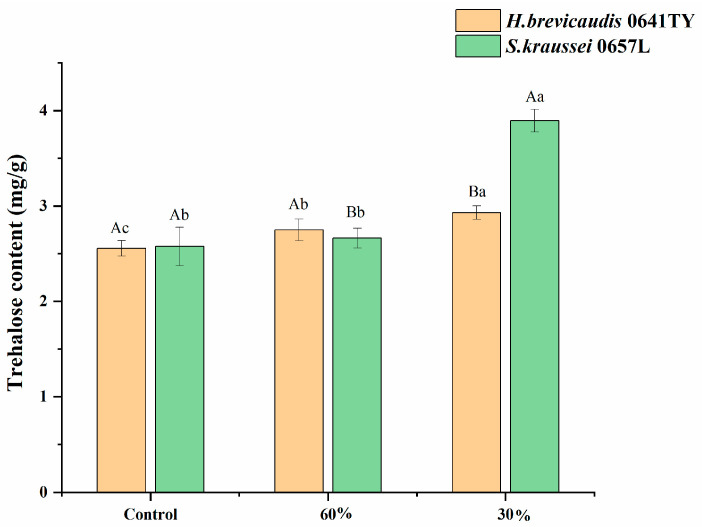
Trehalose content in *H. brevicaudis* and *S. kraussei* after exposure to different humidity levels (control means IJs were stored in distilled water, RH 100%; the measured trehalose content was the content of all IJs after low-humidity stress). Different lowercase letters indicate significant differences between levels of relative humidity for the same nematode strain at a 0.05 significance level. Different capital letters indicate significant differences between nematode strains under the same relative humidity at a 0.05 significance level (Tukey’s test).

Trehalose. Trehalose acts as a critical osmoprotectant, accumulating under desiccation stress to stabilize cellular structures. Our results demonstrated that low-humidity stress induced significant trehalose accumulation in both nematodes, but to different extents (Figure 5). In *H. brevicaudis* 0641TY, trehalose content increased progressively as RH decreased, peaking with the highest value of 2.93 mg/g at 30% RH. This value was significantly higher than that of the control and the 60% RH treatment (*p* < 0.05). Notably, *S. kraussei* 0657L exhibited a much stronger accumulation capacity. Its trehalose content rose sharply with decreasing humidity, reaching a maximum of 3.89 mg/g at 30% RH. Statistical analysis confirmed that this peak value in *S. kraussei* 0657L was not only significantly higher than its own control (*p* < 0.05) but also significantly surpassed the maximum level observed in *H. brevicaudis* 0641TY (*p* < 0.05). This superior ability to synthesize osmoprotectants correlates with the enhanced desiccation tolerance observed in *S. kraussei* 0657L.

**Figure 6:**
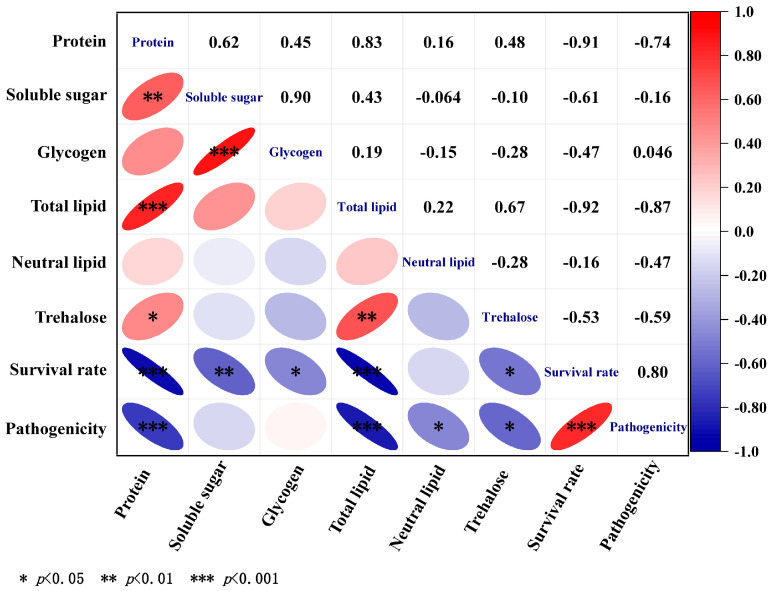
Correlation analysis of energy reserves (mg/g) in *H. brevicaudis* 0641TY with survival rate and pathogenicity. Color code ranges from blue = strong negative correlation (r = −1) to white = no correlation (r = 0) to red = positive correlation (r = +1).

Correlation analysis of energy substances in entomopathogenic nematodes with survival and pathogenicity. Pearson’s correlation coefficient (r) was used to evaluate the correlation between the changes in protein, carbohydrate, and lipid contents in *H. brevicaudis* and its survival rate and pathogenicity (Figure 6). Values greater than 0.8 indicated a strong positive correlation. The results indicated a significantly positive correlation between protein and total lipid (*p* < 0.001, r = 0.83), soluble sugar and glycogen (*p* < 0.001, r = 0.90), soluble sugar and protein (*p* < 0.01, r = 0.62), trehalose and total lipid (*p* < 0.01, r = 0.67), and survival rate and pathogenicity (*p* < 0.001, r = 0.80). In contrast, a significantly negative correlation was observed between survival rate and protein (*p* < 0.001, r = −0.91), survival rate and total lipid (*p* < 0.001, r = −0.92), pathogenicity and protein (*p* < 0.001, r = −0.74), and pathogenicity and total lipid (*p* < 0.001, r = −0.87).

**Figure 7:**
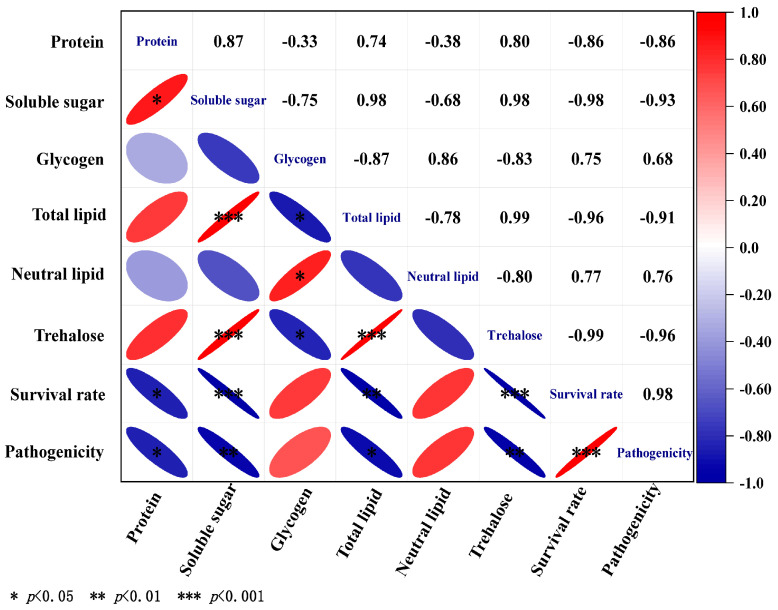
Correlation analysis of energy reserves (mg/g) in *S. kraussei* 0657L with survival rate and pathogenicity. Color code ranges from blue = strong negative correlation (r = −1) to white = no correlation (r = 0) to red = positive correlation (r = +1).

Compared to *H. brevicaudis* 0641TY, *S. kraussei* 0657L exhibited stronger correlations between the measured parameters (Figure 7). There were positive correlations between soluble sugar and trehalose (*p* < 0.001, r = 0.98), soluble sugar and total lipids (*p* < 0.001, r = 0.98), glycogen and neutral lipid (*p* < 0.05, r = 0.86), total lipid and trehalose (*p* < 0.001, r = 0.99), and the survival rate and pathogenicity (*p* < 0.001, r = 0.98). In contrast, we found a significant negative correlation between soluble sugar and survival rate (*p* < 0.001, r = −0.98), soluble sugar and pathogenicity (*p* < 0.01, r = −0.93), glycogen and trehalose (*p* < 0.05, r = −0.83), glycogen and total lipid (*p* < 0.05, r = −0.87), total lipid and survival rate (*p* < 0.01, r = −0.96), trehalose and survival rate (*p* < 0.001, r = −0.99), and trehalose and pathogenicity (*p* < 0.01, r = −0.96). These findings demonstrated that the energy substances in nematodes were closely related to their survival rate and pathogenicity under varying humidity levels, and accumulation of these substances enhanced their adaptability to stress.

### 3.4. Analysis of DEGs in S. kraussei 0657L Under Varying Humidity

**Figure 8:**
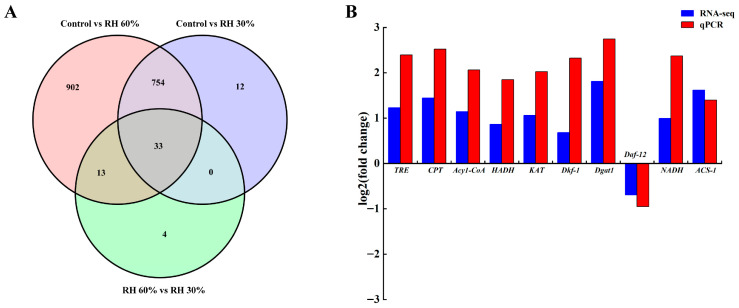
Venn diagram of differentially expressed genes of *S. kraussei* 0657L after low humidity and validation of RNA-seq data and RT-qPCR. (**A**) The number of differential genes of *S. kraussei* 0657 L under 60% and 30% relative humidity stress; (**B**) validation of RNA-seq data by RT-qPCR. The x-axis represents the specific gene selected for validation, and the y-axis indicates the relative expression levels (log_2_ fold change). Data are expressed as mean ± SE.

To understand the drought resistance mechanisms of the drought-tolerant strain *S. kraussei* 0657L, transcriptomic analysis was conducted under varying relative humidity conditions. The analysis identified 2610 DEGs (Figure 8). At 60% relative humidity (RH), 1026 genes were up-regulated and 496 were down-regulated, whereas at 30% RH, fewer DEGs were observed, with 660 up-regulated and 248 down-regulated. Additionally, the RT-qPCR results showed a consistent trend with the RNA-seq data, confirming the reliability of the transcriptomic analysis (Figure 8B).

To further interpret the function of the genes, the KEGG (Kyoto Encyclopedia of Genes and Genomes) database was utilized for pathway annotation analysis of the DEGs (Figure S1). The analysis revealed that under low-humidity stress, DEGs were enriched in pathways such as lysosome and fatty acid metabolism, including fatty acid degradation. At 30% relative humidity, the DEGs enriched in the glycerophospholipid metabolism pathway were up-regulated (Figure S1B). These results suggested that the metabolism of energy substances in *S. kraussei* 0657L played a crucial role in the adaptation of IJs to low-humidity stress.

The Gene Ontology (GO) database comprehensively annotates the functional properties of genes and gene products in organisms. The DEGs in *S. kraussei* 0657L under varying relative humidity conditions were analyzed and categorized into three main groups: Cellular Component (CC), Molecular Function (MF), and Biological Process (BP) (Figure S2). Among the DEGs, the largest proportion was enriched in BP, while the smallest proportion was associated with MF. The analysis revealed that the proportion of DEGs enriched in the three categories remained relatively consistent across different relative humidity levels. Notably, a significant number of DEGs were enriched in metabolic processes within the BP category.

### 3.5. Protein Interaction Network Analysis of DEGs of S. kraussei 0657L Under Different Humidity

**Figure 9:**
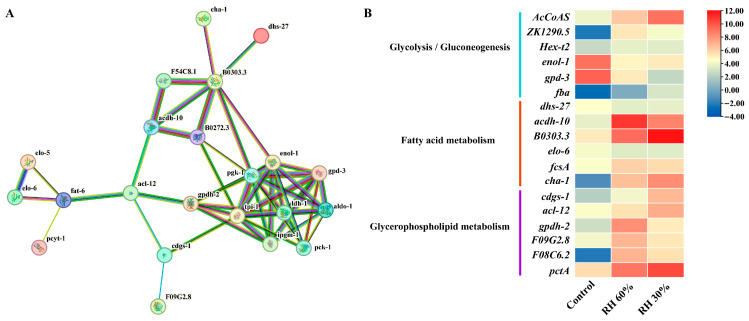
Protein–protein interaction (PPI) network of DEGs related to energy metabolism and expression pattern of selected key genes. (**A**) PPI network of DEGs related to glycolysis/gluconeogenesis, fatty acid metabolism, and glycerophospholipid metabolism, where each node represents all the proteins produced by a single, protein-coding gene locus. Edges represent protein–protein associations: turquoise: from curated databases; magenta: experimentally determined; lime green: gene neighborhood; red: gene fusions; blue: gene co-occurrence; olive drab: text mining; gray: co-expression; light slate blue: protein homology. (**B**) Expression patterns of key genes in three metabolic pathways under different humidity.

A total of 39 DEGs related to energy metabolism (glycolysis/gluconeogenesis 17, fatty acid metabolism 14, and glycerophospholipid metabolism 8) were screened using transcriptomic data with log2 fold change and FPKM as criteria. Using the STRING database (version 12.5, https://string-db.org/, 28 October 2025), the regulatory network of the DEGs was predicted (Figure 9A). This network analysis enabled us to deduce the potential function of DEGs in the energy metabolism. The results demonstrated significant interactions among genes within the three major energy metabolism pathways, with the strongest interactions observed in the glycolysis/gluconeogenesis pathway. Notably, acl-12 acts as a hub node linking the three main energy metabolism pathways. These findings further suggested that IJs respond to low-humidity stress by regulating different forms of energy substances in vivo. Additionally, we analyzed the expression patterns of several genes involved in different metabolic pathways under varying humidity (Figure 9B). The results revealed that the expression levels of most genes, including *acdh-10*, *B0303.3*, *fcsA*, and *AcCoAS*, were significantly up-regulated in response to low-humidity stress, indicating their crucial role in enhancing the resistance of IJs to low humidity conditions.

## 4. Discussion

As the dominant nematode species in Gansu Province, in China, *S. kraussei* 0657L and *H. brevicaudis* 0641TY demonstrated significant potential to adapt to local climatic conditions. Among them, *S. kraussei* 0657L exhibited stronger resistance and greater tolerance to low-humidity stress compared to *H. brevicaudis* 0641TY. These findings align with the results of Yaari et al. [16], who reported that three nematode strains with strong stress resistance had higher survival rates and pathogenicity under thermal or osmotic shock. The enhanced stress adaptability of nematodes serves as the foundation for their successful implementation in insect prevention and control. Notably, stress resistance during the infective juveniles (IJs) stage is often superior to that at other developmental stages [28]. During the infection process, energy reserves in the larvae serve as the sole energy source. Under environmental stress, nematodes depend on these energy reserves to survive and adapt [35]. Thus, understanding the responses of energy substances under low-humidity stress is crucial for expanding our knowledge of nematode biology and their defense mechanisms.

Our results suggest that energy reserves play a critical role in supporting the survival and infectivity of nematodes during the dauer stage. Based on the content levels and consumption patterns observed in this study, the contribution of these reserves follows the order: lipids > protein > sugar. Among sugars, the hierarchy was as follows: soluble sugar > glycogen > trehalose. Consistent with observations in other nematodes, EPNs have evolved specialized mechanisms to enhance survival in various soil environments characterized by differing osmotic pressures, temperatures, textures, and chemical compositions [36]. Additionally, metabolic activity during the infection stage is typically suppressed compared to other life stages [37]. This indicates that non-essential metabolic processes are downregulated to redirect limited resources toward essential survival and infection functions [38].

Exposure to varying humidity levels led to an increase in protein content in *H. brevicaudis* 0641TY and *S. kraussei* 0657L, with the increase in *S. kraussei* being significantly higher than in *H. brevicaudis* 0641TY. High levels of soluble sugar have also been shown to enhance stress resistance in nematodes [39]. As a drought-resistant strain, *S. kraussei* 0657L exhibits a more stable ability to accumulate sugar [16]. Dry conditions and high-temperature stress in three *Steinernema* strains resulted in the downregulation of more genes in nematodes with stronger tolerance, whereas less tolerant strains showed up-regulation of more genes. In *Caenorhabditis elegans*, regulatory genes in osmotic pressure, such as *fat-3*, *osm-9*, and *kin-29*, have been identified to play a role in chemical perception [40]. These findings suggest that the down-regulation of genes in *S. kraussei* 0657L after exposure to low-humidity stress may lead to the closure of specific metabolic pathways. However, the mechanism underlying this reduction in energy need demand requires further exploration.

The results also showed that glycogen content in the two nematodes initially increased and then decreased as relative humidity (RH) decreased. However, glycogen levels were higher in *H. brevicaudis* 0641TY compared to the highly drought-resistant *S. kraussei* 0657 L. This aligns with findings by Patel and Wright [41], who observed high glycogen levels in *S. carpocapsae* under environmental stress. These results suggest that in the less tolerant *H. brevicaudis* 0641TY, more genes may be mobilized to cope with low-humidity stress. Early studies also reported that nematode metabolism shifts from glycogen to trehalose during dehydration [42,43]. In our study, exposure to low-humidity stress resulted in an increase in trehalose levels in both *H. brevicaudis* 0641TY and *S. kraussei* 0657L, with trehalose levels being significantly higher in *S. kraussei* 0657L. Trehalose, an osmoprotectant synthesized under dry conditions, is considered a marker for nematodes entering dehydration [44,45,46]. Our findings also revealed that while glycogen content decreased in both species with decreasing RH, trehalose content increased. The observed depletion of energy reserves is consistent with the well-established fact that IJs are non-feeding during the infection stage, although interconversion among different energy reserves may still occur. For instance, lipid metabolism can lead to an increase in sugar content [47]. This transformation occurs through the activation or silencing of specific genes, such as glycerokinase (GK), which plays a critical role in the glycerolipid metabolism pathway in nematodes. Under low humidity conditions, GK expression increases, along with the upregulation of the glycolysis or gluconeogenesis pathways. This process catalyzes the conversion of glycerol into glycerol-3-phosphate and dihydroxyacetone phosphate, which are subsequently converted into trehalose [31,47,48].

Lipids serve as the primary form of energy storage in nematodes, with neutral lipids comprising a fraction of the total lipid content. In this study, the total lipid content in *S. kraussei* 0657L was generally lower than that in *H. brevicaudis* 0641TY. Although there was no significant difference in neutral lipid levels between the two strains under control conditions, their metabolic responses to desiccation stress diverged. Specifically, the neutral lipid content in *S. kraussei* 0657L decreased significantly as RH dropped to 30%. This depletion suggests that *S. kraussei* 0657L actively mobilizes its stored neutral lipid (like triacylglycerols) to generate energy or metabolic precursors to cope with severe stress. Free-living IJs are known to utilize fatty acids as their primary energy source, a process also observed in other parasitic nematode species [49,50]. Additionally, the hydrolysis of neutral lipids can provide essential fatty acids and energy for survival [51]. Transcriptomic studies in *C. elegans* have also identified DEGs related to lipid metabolism and stress response that regulate the allocation of energy reserves [52,53]. Therefore, the efficient utilization of neutral lipid in *S. kraussei* 0657L likely plays a pivotal role in its superior drought resistance.

The type and level of energy substances play crucial roles in the IJs before they locate their next host. The response to stress is reflected not only in the adaptability of nematodes’ biological properties but also directly related to their behavioral characteristics under stress [10]. This suggests a correlation between energy substances and drought resistance in nematodes. Our results showed that trehalose and soluble sugar were negatively correlated with the survival rate and pathogenicity of *S. kraussei* 0657L, indicating that these substances may have been consumed during the infection process. However, the positive correlation between trehalose and soluble sugar suggests that they may also be converted into one another. On the other hand, *S. kraussei* 0657L accumulated a significant amount of trehalose, which was found to be significantly negatively correlated with survival rate and pathogenicity. Tali et al. found that exposure to low-humidity stress activated the early response gene, GK, in *S*. *feltiae* IS-6, which is associated with the metabolic pathway and led to the accumulation of high levels of trehalose [31]. These findings suggest that the catabolism of lipids and other reserves under low-humidity stress facilitates the synthesis of osmoprotectants. However, although *H. brevicaudis* 0641TY consumed protein and lipids during stress, its accumulation of osmoprotectants (such as trehalose) appeared insufficient to provide adequate protection against desiccation compared to *S. kraussei* 0657L. Furthermore, a stronger correlation between energy reserves and biological characteristics (survival rate and pathogenicity) was observed in *S. kraussei* 0657L than in *H. brevicaudis* 0641TY. Additionally, *S. kraussei* 0657L exhibited more efficient interconversion among energy reserves, suggesting that this species possesses superior metabolic adaptability to low-humidity stress.

The transcriptome data of *S. kraussei* 0657L, a species with strong drought resistance, revealed that more DEGs were enriched in the glycolysis/gluconeogenesis, fatty acid metabolism, and glycerophospholipid metabolism pathways. Using the STRING database, the protein interaction network of these DEGs within energy metabolic pathways was analyzed. The results demonstrated that these pathways were interconnected. For instance, genes such as *gpdh-2*, *acl-12*, *fat-6*, and *acdh-10* were differentially expressed across multiple metabolic pathways and exhibited close relationships. In *C. elegans*, glycogen storage degrades rapidly under hyperosmotic stress, leading to the accumulation of glycerol through the transcriptional upregulation of glycerol-3-phosphate dehydrogenase (*gpdh-1* and *gpdh-2*) [54]. As a key enzyme in the synthesis of dihydroxyacetone phosphate, *gpdh* plays an essential role in glycolysis/gluconeogenesis [55]. These findings further suggest a dynamic conversion relationship between energy substrates in IJs, supporting their drought tolerance. However, the specific functions of these genes and the mechanisms underlying these transformations require further investigation.

Glycogen, an important energy storage molecule in aged IJs, is also closely associated with the decline in their infectivity. This observation aligns with findings by Tali et al., who reported that juveniles with different infection strategies consume different energy substances. For instance, cruiser species utilize more energy reserves during infection compared to other juvenile types [22,56]. Our results demonstrated a negative correlation between protein and total lipid levels in IJs as well as between their survival rate and pathogenicity. Generally, *Steinernema carpocapsae* are characterized as ambush foragers, whereas *Heterorhabditis bacteriophora* display traits typical of cruising foragers, such as actively moving through the soil to search for hosts and responding strongly to host-produced cues [57]. Since cruise foraging is more energetically costly than ambushing, ambush-foraging nematodes are expected to have lower metabolic rates and extended survivorship compared to cruisers. Previous studies [13] have observed that nematodes with different infection strategies adopt distinct energy consumption patterns. For instance, it has been reported that during the ambusher stage, nematodes primarily rely on carbohydrates, whereas lipids become the primary energy source during the cruising stage [58]. Furthermore, it has been suggested that lipid-depleted *S. carpocapsae* can maintain high levels of infectivity to insect larvae [51].

A complex regulatory network operates in stressed nematodes, contributing to their tolerance of environmental stress. However, in this study, we only analyzed the correlation between three types of energy substances and the survival rates and pathogenicity of *S. kraussei* and *H. brevicaudis* after exposure to low-humidity stress. While both nematode species are dominant in arid areas, they exhibit different resistance abilities to low-humidity stress, which must be taken into account when considering their biological characteristics and behaviors. Understanding the response of EPNs to low-humidity stress is crucial for uncovering the basis of their physiological and behavioral adaptations to adverse environmental conditions. Additionally, the mechanisms by which energy substances regulate nematode behaviors, their adaptation to low-humidity stress, and the associated changes in gene functions need to be further explored.

## Figures and Tables

**Table 1:** Primer sequence of qRT-PCR.

Gene	Abbreviation	Forward Primer (5′-3′)	Reverse Primer (5′-3′)
Internal reference	*β—Actin*	TCGGTATGGGACAGAAGGAC	CATCCCAGTTGGTGACGATA
Trehalose	*TRE*	CGGCAGGATTGAGGTGATGA	CGACACAAGCAAGCGGATG
Choline acetyltransferase	*CAT*	AGCAACCTCAAGCGACAAC	ATACCGAGAATGGCGACACT
3-hydroxyacyl-CoA	*HADH*	GCGAGCGAGTAGTGGAGAA	GACTGACACGGAGGCGATA
Fatty acyl-CoA synthetase A	*Acy1-CoA*	GCGTATTGCCATTGTCGGTTA	GTTCTCGTGGTCTTCGTTGATT
Lysine acetyltransferase	*KAT*	AGCAACCTCAAGCGACAAC	ATACCGAGAATGGCGACACT
Serine/threonine-protein kinase dkf-1	*Dkf-1*	GGAAGCGGATGCCTCTGTA	CGGTTGCCTCTGATGTGTTC
Glycerol-3-phosphate dehydrogenase 1	*GPD-1*	GCGTATTGCCATTGTCGGTTA	GTTCTCGTGGTCTTCGTTGATT
NAD(+) hydrolase	*NADH*	GATTCAGTTCAGGAAGGCAGTT	TGTCATCGGCGGAAGATAGTAA
Acetyl coenzyme A synthetase	*ACS-1*	AAGACTGACGACCGCTTCC	CGCTCTGCTCACTTACATACG

**Table 2:** LT_50_ value of EPNs under different relative humidity. * Time (min) required for 50% mortality of nematodes after low humidity; ^a^ upper and lower limit of the 95% confidence level (CI).

EPNs	LT_50_ *		95% CI ^a^	Slope ± Standard Errors	Correlation Coefficient	Regression Equations
	RH %	Mean Value				
*S. kraussei* 0657L	60	111.847	104.964~119.202	−0.031 ± 0.010	0.886	Y = −0.031X + 3.462
	30	74.503	71.086~77.904	−0.048 ± 0.019	0.907	Y = −0.048X + 3.606
*H. brevicaudis* 0641TY	60	75.299	71.486~78.894	−0.038 ± 0.014	0.948	Y = −0.038X + 2.875
	30	67.423	62.656~71.944	−0.052 ± 0.019	0.868	Y = −0.052X + 3.511

**Table 3:** The pathogenicity of EPNs to *Galleria mellonella* under low-humidity stress. Values with different letters indicate significant differences in pathogenicity under the same infection duration and different relative humidity.

EPNs	RH %	Corrected Mortality %			
		32 h	40 h	48 h	56 h
*S. kraussei* 0657L	Control	55.56 ± 5.57 a	91.67 ± 4.81 a	100 ± 0.00 a	100 ± 0.00 a
	60	52.80 ± 3.35 ab	72.20 ± 5.56 bc	88.90 ± 2.80 a	94.45 ± 2.77 a
	30	22.23 ± 2.77 c	27.78 ± 2.77 d	55.57 ± 3.63 b	66.67 ± 1.67 b
*H. brevicaudis* 0641TY	Control	58.33 ± 4.81 a	88.89 ± 2.77 ab	97.22 ± 8.33 a	97.23 ± 2.77 a
	60	33.33 ± 2.84 bc	66.67 ± 4.81 c	88.90 ± 2.78 a	91.67 ± 0.00 a
	30	25.00 ± 4.79 c	30.56 ± 2.77 d	41.67 ± 4.81 b	58.33 ± 8.33 b

Note: The different lowercase letters in the same column indicate significant differences between levels of relative humidity for the same time at a 0.05 significance level (*p* < 0.05, Tukey’s test). Corrected mortality: the mortality of the *Galleria larvae*, corrected mortality % = (mortality of treatment-mortality of control)/(1-mortality of control) × 100.

## Data Availability

The original contributions presented in this study are included in the article/Supplementary Material. Further inquiries can be directed to the corresponding author.

## Supplementary Materials

The following supporting information can be downloaded at: https://www.mdpi.com/article/10.3390/insects17010022/s1, Figure S1: Kyoto Encyclopedia of Genes and Genomes (KEGG) pathway classification of differentially expressed genes (DEGs) in response to different relative humidity treatments. (A) Comparison between control and 60% relative humidity (RH 60%). (B) Comparison between control and 30% relative humidity (RH 30%); Figure S2: Gene Ontology (GO) functional enrichment analysis of differentially expressed genes (DEGs) in response to different relative humidity treatments. (A) Comparison between control and 60% relative humidity (RH 60%). (B) Comparison between control and 30% relative humidity (RH 30%).
