# Supplementary figures and images for: Response of Energy Reserves in Entomopathogenic Nematodes to Drought-Stress and Expression Analysis of Energy Metabolism-Related Genes in Arid Areas
Source: Insects. 2025 Dec 23;17(1):22. doi: 10.3390/insects17010022 (PMC12841759; doi:10.3390/insects17010022)

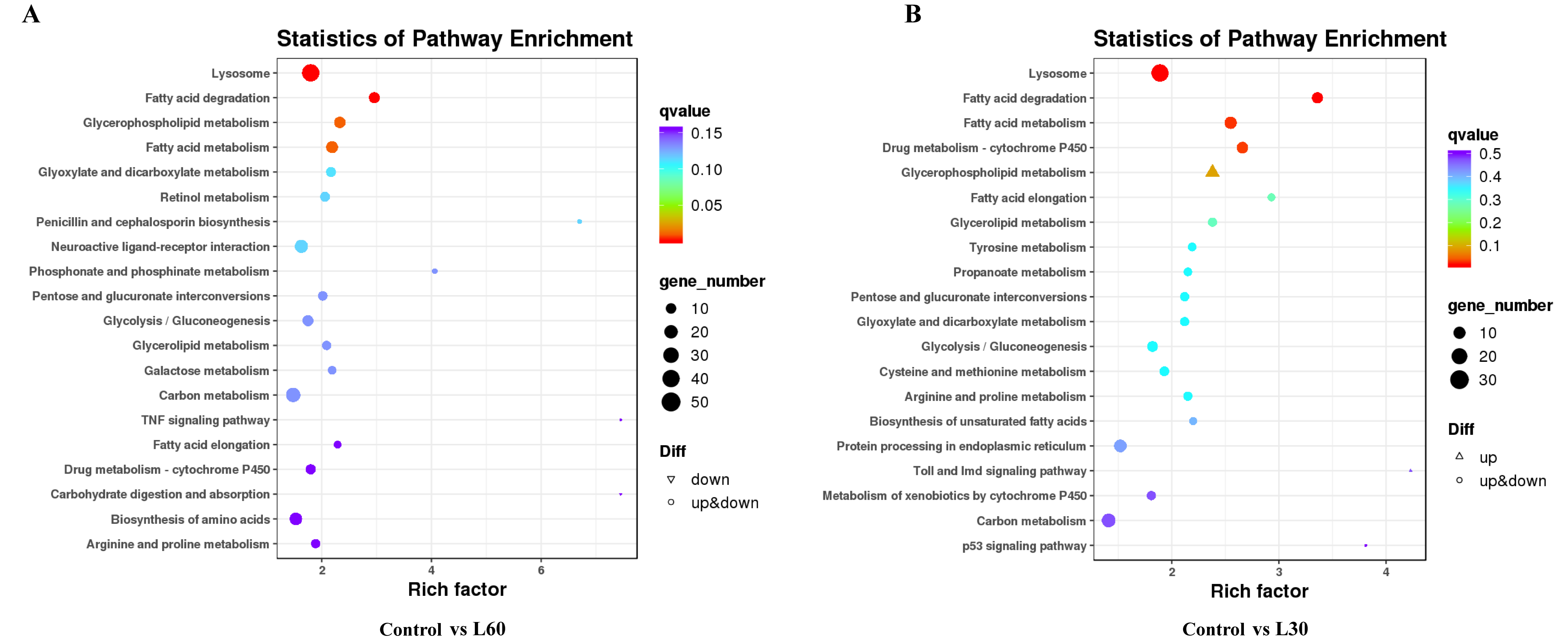

Supplement: Supplementary file 1 [file insects-17-00022-s001.zip › Supplementary figure/Supplementary Figure 1.tif]

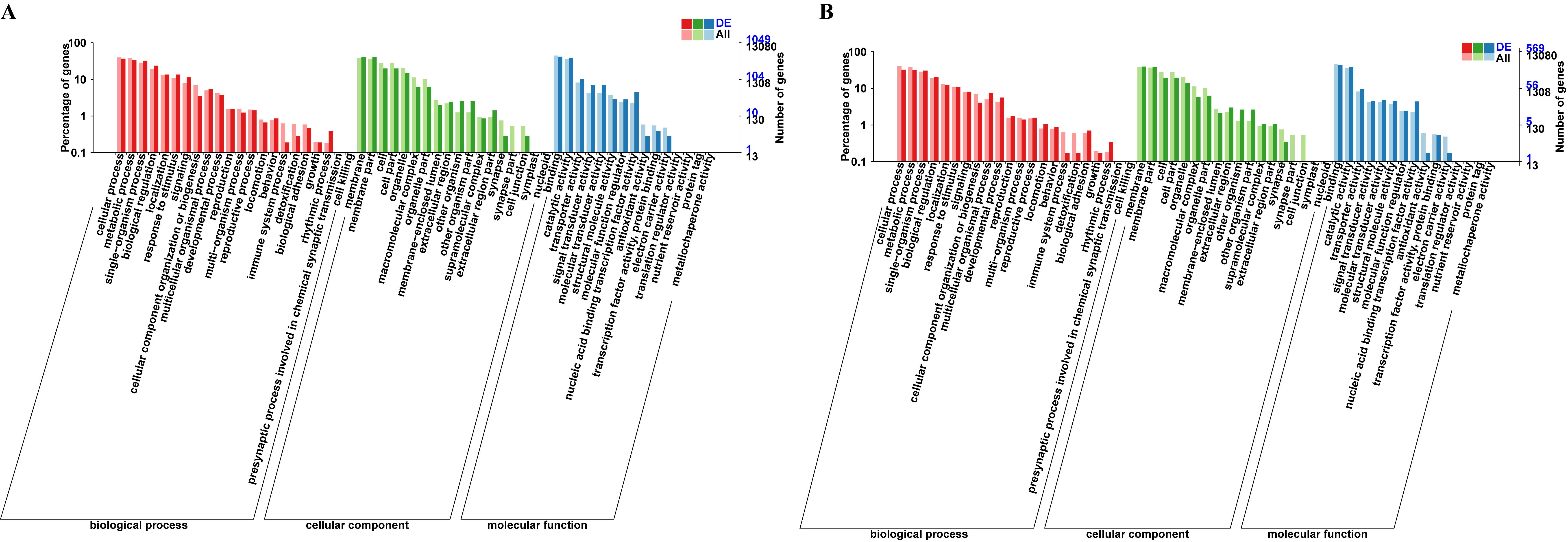

Supplement: Supplementary file 1 [file insects-17-00022-s001.zip › Supplementary figure/Supplementary Figure 2.tif]
